# Case report: Fatal overwhelming post-splenectomy infection in a patient with metastatic angiosarcoma treated with immunotherapy

**DOI:** 10.3389/fimmu.2024.1366271

**Published:** 2024-05-08

**Authors:** Carlos Torrado, Mehmet A. Baysal, Abhijit Chakraborty, Becky L. Norris, Fareed Khawaja, Apostolia M. Tsimberidou

**Affiliations:** ^1^ Department of Investigational Cancer Therapeutics, The University of Texas MD Anderson Cancer Center, Houston, TX, United States; ^2^ Department of Infectious Diseases, Infection Control, and Employee Health, Division of Internal Medicine, The University of Texas MD Anderson Cancer Center, Houston, TX, United States

**Keywords:** OPSI, infection, immunology, immuno-therapy, splenectomy, CTLA4

## Abstract

A patient in his 40s with splenic angiosarcoma metastatic to the liver underwent splenectomy, chemotherapy, and partial hepatectomy before being treated on a clinical trial with CTLA4 and PD1 inhibitors. He had received pneumococcal and meningococcal vaccines post-splenectomy. On week 10, he developed grade 3 immune-related colitis, successfully treated with the anti-tumor necrosis factor-alpha inhibitor infliximab and steroids. After 4 cycles of treatment, scans showed partial response. He resumed anti-PD1 therapy, and 6 hours after the second dose of anti-PD1 he presented to the emergency room with hematemesis, hematochezia, hypotension, fever, and oxygen desaturation. Laboratory tests demonstrated acute renal failure and septicemia (*Streptococcus pneumoniae*). He died 12 hours after the anti-PD1 infusion from overwhelming post-splenectomy infection (OPSI). Autopsy demonstrated non-viable liver tumors among other findings. In conclusion, patients undergoing immunotherapy and with prior history of asplenia should be monitored closely for OPSI as they may be at increased risk.

## Introduction

The role of the spleen is vital for hematological and immunological functions and protects from infections, producing antibodies and filtering bacteria from the bloodstream (1, 2). Splenic trauma and spleen-related diseases often require splenectomy, which results in delayed and impaired antibody production, and other immunological impairments, exposing patients to increased risk of infection and thromboembolism (1–7). Prior to splenectomy, patients should be vaccinated against encapsulated bacteria; this includes *Streptococcal pneumoniae, Haemophilus influenzae* and *Neisseria meningitidis.* Despite best practices, patients may still develop overwhelming post-splenectomy infection (OPSI) due to fulminant bacterial infections.

Here, we present a patient with a history of metastatic splenic angiosarcoma, who had a splenectomy followed by vaccination against encapsulated bacteria and was treated with immunotherapy that was complicated with OPSI.

## Case presentation

**Figure 1:**
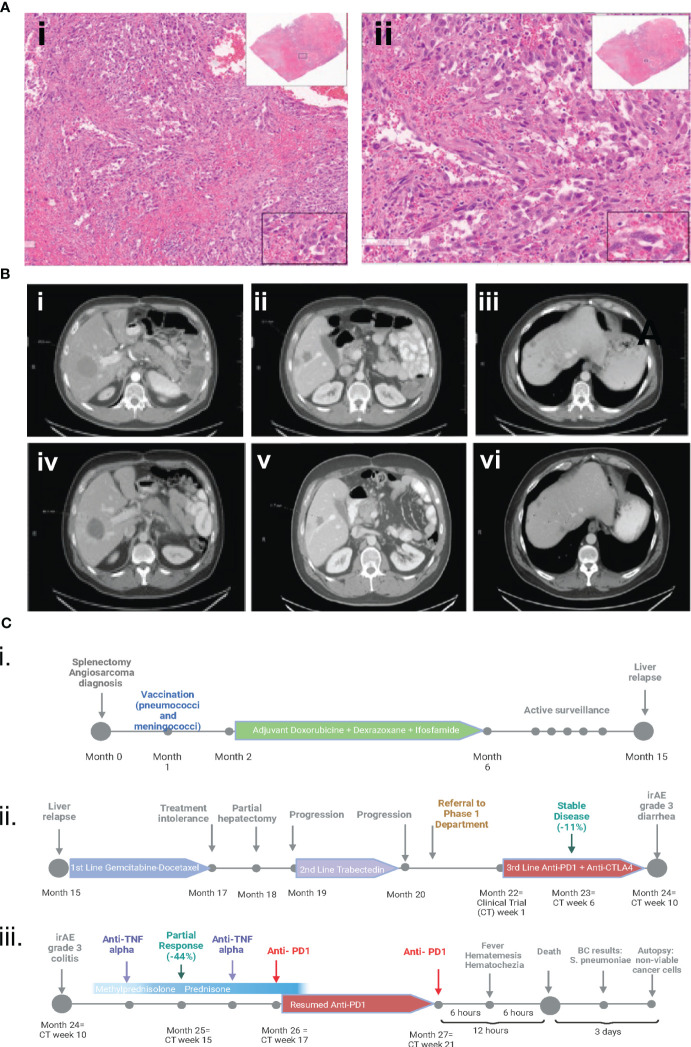
**(A)** Hematoxylin and eosin pathology slides from initial splenectomy. The fusocellular morphology, irregular nuclear atypia with enlarged nuclei, and spindle cell areas are indicative of splenic angiosarcoma with epithelioid features. (i) at 6x magnification and (ii) at 14x magnification. **(B)** CT scans indicating a partial response to immunotherapy. Images (i) to (iii) depict baseline CT scans before the initiation of anti-CTLA4 and anti-PDL1 therapy. Images (iv) to (vi) show partial response to treatment on restaging CT scans after 4 cycles. **(C)** Clinical course timeline of the case. Part i. encompasses the initial diagnosis involving splenectomy, followed by vaccination and adjuvant therapy. Part ii. delineates the three successive lines of metastatic therapy. Part iii. details the treatment of grade 3 immune-related diarrhea, its management with infliximab and steroids, the resumption of anti-PD1 therapy, and the subsequent fatal OPSI. **Figure 1C** (i. ii. iii) was created with biorender.com.

A man in his early 40s visited the emergency center due to abdominal pain and difficulty breathing. A computed tomography (CT) scan of the chest, abdomen and pelvis demonstrated an enlarged spleen and multiple large not well-enhanced masses. The patient underwent splenectomy. Pathological evaluation demonstrated high-grade splenic angiosarcoma with epithelioid features (**Figure 1A**). One month later, CT imaging of the chest, abdomen and pelvis demonstrated a 5-mm left lower lung nodule, moderate pericardial effusion, peripancreatic fluid collection with air (3.2 x 2.2 cm), and significant pancolitis. A flexible sigmoidoscopy demonstrated a severe extrinsic stricture in the splenic flexure that required partial colectomy. This procedure was complicated by an intra-abdominal abscess, treated with antibiotics, and a *Clostridium difficile* infection, treated with oral vancomycin.

One month after splenectomy, the patient received the following vaccines: PREVNAR^®^ 13 (pneumococcal 13-valent conjugate vaccine), MCV-4 (conjugate vaccine against *Neisseria meningitidis*), and BEXSERO (meningococcal group B vaccine). Subsequently, he was treated with doxorubicin, dexrazoxane, ifosfamide, and mesna (x6 cycles). He was placed on watchful waiting, and 10 months later, positron emission tomography (PET)/CT imaging revealed a hepatic lesion that was biopsy-proven to be metastatic angiosarcoma. The patient was then treated with gemcitabine and docetaxel (x2 cycles), which was discontinued because of poor tolerance. Hepatectomy of the solitary lesion was performed; however, a subsequent PET/CT scan demonstrated progressive disease (increase in number and size of liver metastases). Consequently, he received trabectedin (x2 cycles), which was discontinued because of progressive disease in the liver. The patient was then referred to The University of Texas MD Anderson Cancer Center.

Molecular profiling of the liver (performed at MD Anderson Cancer Center) demonstrated multiple molecular alterations, high tumor mutational burden (TMB) (14.7 mut/MB; 93rd percentile) and programmed death-ligand 1 (PDL1) expression in ~ 50% of tumor cells (strong membranous staining) (**Table 1**). The patient began a clinical trial of combination therapy with a CTLA4 inhibitor (every 6 weeks) and a PD-1 inhibitor (every 2 weeks). After 6 weeks of treatment, CT scans demonstrated stable disease (RECIST, decrease in tumor measurements by 14%) and the patient continued treatment on protocol. At week 10 of treatment, the patient experienced grade 3 diarrhea, that required hospitalization. Infectious disease work-up demonstrated a positive *Streptococcus* group A rapid test and nasopharyngeal swab polymerase chain reaction was positive for coronavirus OC43 (common cold). The remaining respiratory viral panel, stool, and blood cultures were negative. Polymerase chain reaction testing for *Clostridium difficile* was negative. The patient was assessed by the Infectious Diseases team while he was hospitalized. He was treated for strep throat infection initially with ceftriaxone and later with amoxicillin (functionally asplenic patient). His vaccines (PREVNAR^®^ 13, MCV-4, and BEXSERO) were confirmed to be up to date (administered within 2 years). CT scans showed mild distention of the colon and no other significant findings. He underwent sigmoidoscopy and was found to have scattered mild inflammation characterized by erosions and erythema in the rectum. Biopsy of the rectum demonstrated rectal mucosa with focal acute cryptitis and reactive changes. The patient was treated with steroids and infliximab and the diarrhea resolved (steroid tapering x1 month, and the second dose of infliximab was administered 3 weeks after the first dose).

**Table 1:** Molecular profile.

**Genomic Variants-** **146-gene panel**	**TSC2** p.R751* Stop gain – LOF **POT1** p.R117C Missense variant – LOF **TP53** p.R213* Stop gain – LOF **RB1** p.R552* Stop gain – LOF **CIC** p.R528* Stop gain – LOF **MSH6** Copy number loss
**Immunotherapy Markers**	**Tumor Mutational Burden,** High, 14.7 m/MB; 93rd percentile **Microsatellite Instability Status**, Stable **PD-L1 expression**, 50%
**Variant of Unknown Significance**	**UBE2T** c.206G>A p.R69Q Missense variant **MTHFR** c.1159G>A p.G387S Missense variant **LZTR1** c.2131G>A p.G711R Missense variant **DNMT3A** c.97C>T p.R33C Missense variant **AXIN2** c.2141G>A p.R714Q Splice region variant **BMPR1A** c.766G>A p.E256K Missense variant **RBM27** c.2448del p.K816fs Frameshift **MET** c.1277G>A p.R426H Missense variant **WNK2** c.2920C>T p.R974W Missense variant **PTPN13** c.965G>A p.R322H Missense variant **SRFBP1** c.1256T>A p.V419E Missense variant **TCF3** c.940G>A p.A314T Missense variant **FGFR4** c.1306G>A p.V436M Missense variant **CARD11** c.215G>A p.R72Q Missense variant **PTPRT** c.1352G>A p.G451D Missense variant **GLI2** c.3154G>A p.D1052N Missense variant **FAT1** c.824T>C p.I275T Missense variant

LOF, loss of function.

CTLA4 inhibitor treatment was discontinued on week 12 (he received a total of 2 doses) and the patient continued treatment on protocol with the PD-1 inhibitor alone. At week 17, the patient was feeling well, and his laboratory tests were normal. CT scans revealed a partial response (RECIST, decrease in tumor measurements by 44%) (**Figure 1B**), and he started cycle 5 of treatment with the PD-1 inhibitor in the outpatient setting. Six hours after infusion of the PD-1 inhibitor, the patient experienced hematemesis and hematochezia and was transferred to the emergency room, where he was found to be unresponsive. His temperature was 39.7° C, blood pressure 88/68 mmHg, heart rate 90/min, respirations 12/min, and O_2_ saturation 50%. He received vasopressors and was intubated. Laboratory tests showed a creatinine level of 3.25 mg/dL (normal value, 0.7-1.3 mg/dL) and a troponin I level of 229 ng/dL (normal value, <59 ng/dL). The patient’s white blood cell count was normal. The patient’s condition rapidly worsened, and he died within 12 hours after infusion of the PD-1 inhibitor. Blood cultures drawn prior to his death were positive for *Streptococcus pneumoniae*, confirming septicemia.

An autopsy performed 3 days after the patient’s death revealed multiple necrotic tumor nodules with *non-viable* neoplastic cells present in the liver, extensive bowel adhesions, pulmonary edema, and left ventricular dilatation. The small and large bowel was markedly autolyzed, but no histopathologic findings were identified. Blood cultures at autopsy confirmed the presence of *Streptococcus pneumoniae* (**Figure 1C**).

## Discussion

This is the first case report of a patient treated with immunotherapy who had undergone splenectomy and died from an OPSI, as evidenced by clinical presentation, laboratory findings and autopsy results. Immunotherapy may increase the risk of infections or modify the coverage provided by vaccination post-splenectomy. Additionally, the patient’s immune-related grade 3 diarrhea, which was assumed to be colitis, was treated with the tumor necrosis factor-alpha (TNF-α) inhibitor infliximab and steroids, which likely further enhanced the immunosuppression (8, 9).

OPSI is a rare condition (prevalence rate, 0.1%-0.5%). It is mainly caused by encapsulated bacteria, such as *Streptococcus pneumoniae* (10), *Escherichia coli* (11), *Neisseria meningitidis* (12)*, Haemophilus influenzae (type b)* (13), *Capnocytophaga* spp (14), and *Bordetella holmesii* (15). OPSI presents with nonspecific flu-like symptoms, such as nausea, vomiting, fever, and unconsciousness, followed by rapid deterioration to full-blown fulminant septic shock within 24-48 hours. It is associated with a high rate of mortality (38-70%) (10, 11); however, this risk can be mitigated to less than 10% when well-informed patients promptly seek medical attention (16). The overall increased risk of OPSI remains throughout the patient’s lifespan, with the first 3 years following splenectomy being the most critical (2, 3).

Immunotherapy is known to increase the risk of infection by causing immunosuppression (8). Investigators found that, among a retrospective cohort of 111 patients with various solid malignancies treated with immunotherapy, the overall serious infection (requiring hospital admission and/or intravenous antibiotics) rate was 14% (17). Additionally, the use of steroids was associated with an increased risk of serious infection (75% when steroids were used versus 28.4% with no steroid use, p=0.0003) (17). Furthermore, a meta-analysis including 21,451 patients demonstrated that the risk of serious infection is higher in patients who received chemo-immunotherapy compared to chemotherapy alone (relative risk [RR] = 1.52, 95% CI 1.17–1.96; *P* < 0.01), indicating an additional risk of infection posed by immunotherapy in cancer patients (18).

In addition to splenectomy and immunotherapy, the use of steroids and anti-TNF-α therapy may have contributed to the development of OPSI in our patient. TNF-α plays a pivotal role in the recruitment of neutrophils, eosinophils, and macrophages to infection sites (19, 20). A meta-analysis has reported that in patients with rheumatoid arthritis treated with anti-TNF-α therapy, the odds ratio of infection was 2.0 (95% CI, 1.3–3.1) compared with patients treated with placebo (21). It is plausible that in our patient, anti-TNF-α and steroids for the treatment of grade 3 immune-related diarrhea contributed to immunosuppression associated with splenectomy, angiosarcoma, and immunotherapy. Notably, pneumococcal vaccination is recommended prior to anti-TNF-α initiation (22), and our patient’s vaccination was up to date. However, 30% of pneumococcal infections in OPSIs stem from serotypes not covered by the vaccine (23). Our patient had been fully vaccinated post-splenectomy, 2 years before receiving anti-TNF-α. It was determined that no further vaccination was required. However, further research and guidelines should be developed regarding the optimization of vaccination before anti-TNF-α to prevent OPSI.

This case report highlights the need to prevent OPSI, particularly in patients treated with immunotherapy. In a review of 42 splenectomized patients with OPSI (24), investigators found that OPSI occurred up to 59 years after splenectomy, the mortality rate was 45%, and pneumococcal infection was noted in 37 of the 42 episodes. Only 12 patients had received pneumococcal vaccination, 22% had received chemoprophylaxis since splenectomy, and only 1 had a medical alert card (24). In another study, the risk of OPSI persisted for 5 years among asplenic patients (25). Though 92% received vaccinations, including *Haemophilus influenzae type B, meningococcal C*, and *pneumococcus*, the investigators concluded that there was a compelling need for enhanced management through standardized protocols and heightened awareness to mitigate OPSI risks. Other investigators reviewed 162 adult splenectomized patients who were observed for 25 years, and 4% experienced OPSI (median age, 37 years), most commonly caused by *Streptococcus pneumoniae*. Despite preventive measures, the risk of OPSI persisted, indicating the challenges in managing this condition. Others (26) have also reported the seriousness of OPSI and its high mortality rate, even with proper vaccination and education, stressing the need for lifelong vigilance. A meta-analysis (27) that explored immunization coverage among asplenic patients revealed suboptimal adherence to vaccine recommendations, necessitating intensified public health initiatives to bolster vaccination compliance. These align with the overarching emphasis on education and prevention, crucial components in the multifaceted approach to mitigating OPSI risk.

Investigators have provided mechanistic insights regarding T-cells in splenectomized patients after vaccination (28). Comparing vaccine-specific memory B- and T-cells, they found reduced numbers of memory IgG B cells and undetectable levels of memory IFN-γ T-cells, indicating the role of the spleen in generating an effective immune response (28).

Together, these studies highlight persistent challenges in managing OPSI risks in asplenic individuals. Adopting holistic strategies that focus on enhanced patient education, standardized protocols, and continuous monitoring is crucial to reduce OPSI risks effectively. These strategies are supported by our case and the literature, which stress the critical role of patient education, vaccination, and sustained antibiotic prophylaxis in managing OPSI risks. It is essential to administer vaccinations that target encapsulated bacteria in a timely manner and to consistently adopt measures to prevent OPSI complications in this patient population.

Finally, an intriguing finding in this case report is that the patient’s liver metastases had a necrotic appearance on autopsy, indicating that no active angiosarcoma was present despite the PET/CT report stating that there was residual disease. This observation highlights the need to perform PET/CT and, if needed, biopsies of residual tumors evident on CT imaging in patients treated with immunotherapy to determine whether active tumor is present. Treatment should be tailored to individual patients’ needs. Physicians should assess and discuss with the patients the risks and benefits associated with continuation of immunotherapy if no active tumor is present. This is particularly important because the use of anti-PD1 and anti-CTLA4 has been associated with eradication of tumor in patients with various tumor types, including melanoma (29), renal cancer (30), microsatellite-instable colorectal cancer (31), high-grade neuroendocrine carcinoma (32) and prostate cancer (33) (and personal experience). Guidelines should be developed for immunotherapy trials to include PET/CT imaging and biopsies for individual patients who respond to treatment to determine whether further immunotherapy is indicated.

In conclusion, this case highlights the intricate and delicate balance between therapeutic interventions and the potential for life-threatening consequences, especially in patients with cancer who have had a splenectomy and then received immunotherapy. It underscores the critical importance of caution, vigilance, and well-informed decision-making by both clinicians and patients to navigate the risks associated with infectious complications after splenectomy. The current guidelines stress the importance of fully vaccinating patients before undergoing splenectomy. These data emphasize the need for screening patients for a history of splenectomy, which may not always be evident on the patient’s records. This awareness is not merely informative. Caution is needed, urging healthcare professionals and patients to work collaboratively for education, vaccination, antibiotic prophylaxis, and lifelong monitoring of asplenic individuals to prevent OPSI. Prophylactic levofloxacin should be prescribed in case patients develop fever. Immunotherapy related complications may have mitigated the benefits of vaccines. Patients should continuously be assessed for the benefit versus risk associated with immunotherapy, and PET/CT imaging and biopsies should be considered for individual patients who respond to treatment to determine whether further immunotherapy is indicated.

## Data availability statement

The original contributions presented in the study are included in the article/supplementary material, further inquiries can be directed to the corresponding author/s.

## Ethics statement

The patient had signed an informed consent document prior to treatment stating that he was aware of the investigational nature of the study, and he had agreed to publish de-identified data regarding the results associated with the investigational therapy. The information provided in this case report and images has been de-identified and therefore, there are no potentially identifiable data. Written informed consent was obtained from the participant/patient(s) for the publication of this case report.

## Author contributions

CT: Writing – review & editing, Writing – original draft, Visualization, Methodology, Investigation. MB: Formal analysis, Writing – review & editing, Writing – original draft, Visualization, Methodology, Investigation. AC: Writing – review & editing, Writing – original draft, Visualization, Methodology, Investigation, Formal analysis. BN: Data curation, Writing – review & editing, Writing – original draft, Investigation. FK: Investigation, Writing – review & editing, Writing – original draft. AT: Validation, Supervision, Resources, Methodology, Funding acquisition, Data curation, Conceptualization, Investigation, Writing – review & editing, Writing – original draft.
